# Endothelial Na^+^/H^+^ exchanger NHE1 participates in redox-sensitive leukocyte recruitment triggered by methylglyoxal

**DOI:** 10.1186/s12933-014-0134-7

**Published:** 2014-09-30

**Authors:** Syed M Qadri, Yang Su, Francisco S Cayabyab, Lixin Liu

**Affiliations:** Department of Pharmacology, College of Medicine, University of Saskatchewan, 107 Wiggins Road, Saskatoon, Saskatchewan Canada; Department of Physiology, College of Medicine, University of Saskatchewan, 107 Wiggins Road, Saskatoon, Saskatchewan Canada

**Keywords:** Methylglyoxal, NHE1, Oxidative stress, SGK1, Leukocyte recruitment

## Abstract

**Background:**

Excessive levels of methylglyoxal (MG) encountered in diabetes foster enhanced leukocyte-endothelial cell interactions, mechanisms of which are incompletely understood. MG genomically upregulates endothelial serum- and glucocorticoid-inducible kinase 1 (SGK1) which orchestrates leukocyte recruitment by regulating the activation and expression of transcription factors and adhesion molecules. SGK1 regulates a myriad of ion channels and carriers including the Na^+^/H^+^ exchanger NHE1. Here, we explored the effect of MG on SGK1-dependent NHE1 activation and the putative role of NHE1 activation in MG-induced leukocyte recruitment and microvascular hyperpermeability.

**Methods:**

Using RT-PCR and immunoblotting, we analyzed NHE1 mRNA and protein levels in murine microvascular SVEC4-10EE2 endothelial cells (EE2 ECs). NHE1 phosphorylation was detected using a specific antibody against the 14-3-3 binding motif at phospho-Ser^703^. SGK in EE2 ECs was silenced using targeted siRNA. ROS production was determined using DCF-dependent fluorescence. Leukocyte recruitment and microvascular permeability in murine cremasteric microvasculature were measured using intravital microscopy. The expression of endothelial adhesion molecules was determined by immunoblotting and confocal imaging analysis.

**Results:**

MG treatment significantly upregulated NHE1 mRNA and dose-dependently increased total- and phospho-NHE1. Treatment with SGK1 inhibitor GSK650394, antioxidant Tempol and silencing SGK all blunted MG-triggered phospho-NHE1 upregulation in EE2 ECs. NHE1 inhibitor cariporide attenuated MG-triggered ROS production, leukocyte adhesion and emigration and microvascular hyperpermeability, without affecting leukocyte rolling. Cariporide treatment did not alter MG-triggered upregulation of P- and E-selectins, but reduced endothelial ICAM-1 expression.

**Conclusion:**

MG elicits SGK1-dependent activation of endothelial Na^+^/H^+^ exchanger NHE1 which participates in MG-induced ROS production, upregulation of endothelial ICAM-1, leukocyte recruitment and microvascular hyperpermeability. Pharmacological inhibition of NHE1 attenuates the proinflammatory effects of excessive MG and may, thus, be beneficial in diabetes-associated inflammation.

## Introduction

Increased interaction of leukocytes with activated vascular endothelium participates in the inflammatory sequelae of diabetes [1-3]. Excessive levels of the glycolysis metabolite methylglyoxal (MG) *in vivo*, that contribute to increased carbonyl stress, are associated with conditions such as diabetes, renal failure, obesity and metabolic syndrome [4-9]. In diabetes, increased levels of MG are implicated in the pathogenesis of vascular complications such as hypertension [10], impaired microcirculation [11], and thrombosis [12]. Ramifications of pathological MG concentrations include modulation of immune cell functions by stimulation of cytokine induction [13], activation of macrophages [14], and suppression of T-cell functions [15]. MG alters cellular functions by influencing energy and redox balance [16], modulating cytosolic Ca^2+^ [17] and by triggering apoptotic or necrotic cell death [11,18].

MG was shown to influence innate immunity in diabetes by enhancing neutrophil apoptosis and by eliciting the expression of integrins on neutrophils [12]. Recently, MG was shown to stimulate leukocyte-endothelial cell interactions by reducing intravascular rolling velocity and by potentiating adhesion and transendothelial migration of leukocytes through the upregulated endothelial adhesion molecules [19]. MG was shown to induce eNOS uncoupling [20] which supports redox-sensitive leukocyte recruitment and enhanced microvascular permeability during inflammation [21]. However, putative mechanisms of MG-induced leukocyte recruitment are largely unknown.

MG-induced leukocyte recruitment was recently reported to be mediated by signal transduction downstream of endothelial phosphoinositide 3-kinases (PI3K) [22]. MG was shown to temporally activate glycogen synthase kinase 3 (GSK3) and serum- and glucocorticoid-inducible kinase 1 (SGK1) which, in turn, stimulate endothelial nuclear factor-κB (NF-κB) and cyclic AMP response element-binding protein (CREB), two transcription factors that are important in mediating inflammatory responses [22]. The kinase SGK1, ubiquitously expressed and regulated by a myriad of cell stressors [23], is genomically upregulated in endothelial cells in response to MG treatment [22]. SGK1 regulates a wide array of ion channels and carriers including the Na^+^/H^+^ exchangers NHE1 and NHE3 [24-29]. Phosphorylation of NHE1 Ser^703^ by SGK1 is essential for the binding of 14-3-3 protein to NHE1 [25,30] which, in turn, is critical in the activation of this Na^+^/H^+^ exchanger [25,31].

The plasma membrane transport protein NHE1 regulates cellular pH and volume and, thus, participates in a multitude of physiological functions such as proliferation, migration and apoptosis [32]. Endothelial cells express the Na^+^/H^+^ exchanger isoforms NHE1 and NHE2 that regulate functions such as endocytosis [33], apoptosis [34] and blood brain barrier [35-38]. NHE1 is constitutively phosphorylated and additional phosphorylation enhances its activity [39]. Several studies have documented that reactive oxygen species (ROS)-mediated signaling activates NHE1 [24,40]. Na^+^/H^+^ exchange is pharmacologically inhibitable by NHE1-selective acylguanidine-derived compounds such as cariporide (HOE-642) [39,41,42]. Cariporide blunts the phosphorylation of NHE1 [31] and has been widely used to study putative functions of NHE1 *in vitro* and *in vivo* [24,41,43,44]. The influence of MG on NHE1 expression and functions remains elusive.

The present study explores the mechanisms involved in the activation of endothelial NHE1 by MG. Using intravital microscopy, which enables us to directly visualize and determine leukocyte-endothelial interactions with high imaging quality in the microvasculature of anaesthetised mice, we elucidate the effects of pharmacological suppression of NHE1 on leukocyte recruitment and microvascular hyperpermeability elicited by MG.

## Materials and methods

### Mice and intravital microscopy

Male C57BL/6 mice (Charles River, Saint-Constant, QC, Canada) aged 8–12 wk-old were used in this study with the approval of animal protocols from University Committee on Animal Care and Supply (#20070028) at the University of Saskatchewan. Mice were anaesthetised using an i.p. injection of 10 mg/kg xylazine (Bayer, Toronto, ON, Canada) and 200 mg/kg ketamine hydrochloride (Rogar, Montreal, QC, Canada). The mouse cremaster muscle preparation was used to study dynamic leukocyte-endothelial interactions in microvasculature as described [21,22,45,46]. The cremaster muscle microvasculature is considered to be the goldstandard *in vivo* model for intravital microscopy where leukocyte-endothelial cell interactions in the postcapillary venules are readily visualized [47]. Leukocyte rolling flux (cells/min), velocity of rolling leukocytes (μm/sec), and the number of adherent (cells/100-μm venule) and emigrated leukocytes (cells/443 × 286 μm^2^ field) were determined in the cremasteric postcapillary venule (25–40 μm diameter) using video playback analysis [21,22,45,46]. MG-triggered microvascular leakage was determined in postcapillary venules of the cremaster muscle by intravital microscopy measuring fluorescence intensity of FITC-labelled BSA (25 mg/kg i.v.; Sigma) inside and outside the vessel for calculating permeability index as described [21,45]. Where indicated, MG (50 mg/kg; Sigma, Oakville, ON, Canada) and cariporide (20 mg/kg, Sigma) were administered by separate intrascrotal injections or superfusion of exposed cremaster muscle with MG and cariporide at 100 μM and 50 μM, respectively.

### Cell culture and gene silencing

Murine microvascular SVEC4-10EE2 endothelial cell line cells (EE2 ECs; ATCC, Manassas, VA) were cultured as described earlier [22]. Previously, MG-sensitive SGK1 signaling was studied in EE2 ECs [22] and in the present study we elucidate the role of NHE1 in MG-induced leukocyte recruitment in the context of SGK1-dependent activation of NHE1. We, therefore, used EE2 ECs to corroborate our *in vivo* findings. Where indicated, Tempol (300 μM; Sigma), cariporide (50 μM) or GSK650394 (20 μM; Sigma) was added at the specified concentrations. Targeted gene silencing was accomplished by a 48-h transfection of EE2 ECs with siRNA specifically targeting SGK (Santa Cruz) and with siRNA transfection medium and reagent (Santa Cruz) as described previously [48]. The control cells were transfected with negative control scrambled siRNA (Santa Cruz) having no homology to any known RNA sequence.

### RT-PCR

RT-PCR was performed to determine NHE1 and β-actin mRNA expression as described previously [22]. Briefly, RNA was isolated from the cells using RNA isolation kit (Qiagen) and reverse-transcribed using reverse transcription kit (Qiagen). RT-PCR was carried out by SYBR green PCR kit (Qiagen) in an iCycler iQ apparatus (Bio-Rad, Hercules, CA) with primers targeting NHE1 (QT00105413; Qiagen) and β-actin (QT00095242; Qiagen). All PCRs were performed in triplicate and ran for 30 cycles at 95°C for 30 sec, 55°C for 30 sec, and 72°C for 40 sec.

### Immunoprecipitation and immunoblotting

Phosphorylation of NHE1 was determined in NHE1-immunoprecipitated samples using an anti-phospho-(Ser) 14-3-3 protein binding motif antibody [25]. Briefly, EE2 ECs or excised cremaster muscles were harvested using ice-cold IP lysis buffer containing complete protease and phosphatase inhibitor cocktail (Cell Signaling) and the samples were sonicated on ice three times for 5 seconds each. The protein concentration was determined by BCA assay (Sigma). The samples were then aliquoted into two equal portions of 500 μg proteins each. In the first portion, β-actin (Santa Cruz) was detected as described previously [22]. In the second portion, lysates containing 500 μg proteins were incubated overnight at 4°C with 5 μl mouse monoclonal NHE1 antibody (Abcam). Then, immune complexes were mixed with protein G agarose (20 μl of 50% bead slurry, Thermo Fisher Scientific) for 3 h at 4°C and then washed five times with ice-cold IP lysis buffer. The immune complexes were dissociated by adding 20 μl 3× SDS sample buffer and heating for 5 min at 95°C, and then centrifuged for 1 min at 14,000× *g* for removing the agarose beads. The resulting supernatant was separated into two equal portions. One portion of the protein lysate was separated on 10% SDS-PAGE gels and electrotransferred to a nitrocellulose membrane. The membrane was incubated overnight at 4°C with rabbit polyclonal phospho-(Ser) 14-3-3 binding-motif protein antibody (1:1000, Cell Signaling), followed by 2-h incubation with HRP-conjugated goat anti-rabbit secondary antibody (1:1000, Santa Cruz) at room temperature. In the other portion, total NHE1 (1:1000, Abcam) was determined respectively using routine immunoblotting [45]. Where indicated, expression of adhesion molecules was detected in cremaster muscle homogenates as described previously [19,22] using primary antibodies against ICAM-1 (Abcam), P-selectin (LifeSpan Biosciences) and E-selectin (Abcam). Intensity values for the proteins were normalized to β-actin and densitometric quantification of the detected bands was performed using Quantity One® Software (Bio-Rad).

### Determination of ROS production

The levels of ROS production in EE2 ECs were measured as previously described [21]. Following the indicated treatments, EE2 ECs were loaded (30 min, 37°C in the dark) with a membrane-permeable and non-fluorescent 2’,7’-dichlorodihydrofluorescein diacetate (H2DCFDA; 30 μM, Invitrogen) which was oxidized to fluorescent 2’,7’-dichlorofluorescein (DCF) in the presence of ROS. To remove excess probe, the cells were washed with PBS and fluorescence intensity in EE2 ECs was excited at 495 nm and measured at 527 nm emission wavelengths. Data are normalized to the control.

### Confocal imaging

EE2 ECs treated under different conditions were fixed using 4% paraformaldehyde for 30 min, washed twice with PBS, blocked for 1 h with 5% goat serum (Abcam) and then incubated overnight at 4°C with antibodies against ICAM-1 (1:500; eBioscience). After washing, the slides were incubated for 1 h with fluorescence goat anti-rat antibody (Alexa Fluor 488; Invitrogen) and the cells were then washed, permeabilized with 0.1% Triton X-100 and stained with Hoechst 33342 (Invitrogen). The slides were mounted in ProLong® Gold Antifade Reagent (Invitrogen) and observed under a laser scanning confocal microscope (Zeiss, LSM 700). As the antibodies recognize extracellular epitopes of target proteins, the labelled proteins are considered to have surface localization.

### Statistical analysis

Data are shown as arithmetic mean ± SEM. Statistical analysis was made using ANOVA with Tukey’s post-hoc comparison test. *n* denotes the number of different mice, different batches of cremaster muscle or endothelial cells studied in each group. Values of p < 0.05 were considered statistically significant.

## Results

**Figure 1:**
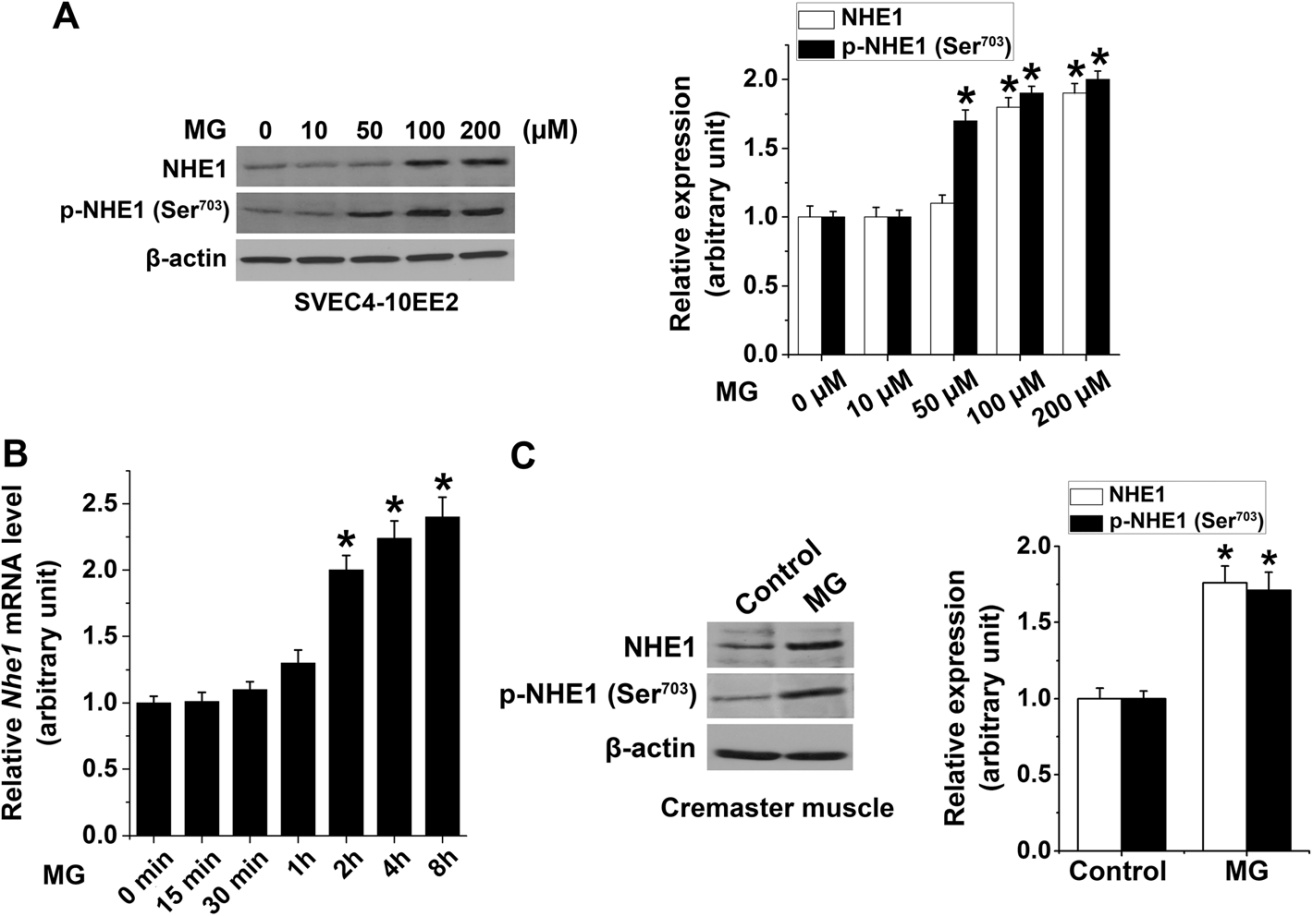
**Methylglyoxal-induced upregulation of NHE1 expression. (A)** Representative original Western blot and means ± SEM (n = 4) showing total NHE1 and phospho-NHE1 (14-3-3 binding motif at p-Ser^703^) determined in MG-treated (0–200 μM for 4 h) EE2 ECs (relative to β-actin). * indicates significant difference (p < 0.05) from 0 μM MG. **(B)** Mean ± SEM of NHE1 mRNA levels (n = 4) determined in MG-treated (100 μM for 0–8 h) EE2 ECs (relative to β-actin). * indicates significant difference (p < 0.05) from 0 h. **(C)** Representative original Western blot and means ± SEM (n = 4) showing total NHE1 and phospho-NHE1 (14-3-3 binding motif at p-Ser^703^; relative to β-actin) determined in excised cremaster muscle 4 h after an intrascrotal injection with saline (Control) or MG (50 mg/kg). * indicates significant difference (p < 0.05) from the absence of MG (Control).

Firstly, we tested whether MG treatment influences endothelial NHE1 protein levels. We found that treatment of EE2 ECs with MG significantly enhanced NHE1 and phospho-NHE1 in a dose-dependent manner, an effect reaching statistical significance at 100 μM MG (NHE1) and 50 μM (phospho-NHE1) respectively (Figure 1A). Next, we determined whether MG influences NHE1 transcription in EE2 ECs. As shown in Figure 1B, treatment of EE2 ECs with MG for 0–8 h significantly enhanced NHE1 mRNA levels, an effect reaching statistical significance 2 h after MG treatment. To corroborate our *in vitro* observations, we demonstrate that an intrascrotal injection of MG for 4 h significantly upregulated total- and phospho-NHE1 levels in cremaster muscle (Figure 1C).

**Figure 2:**
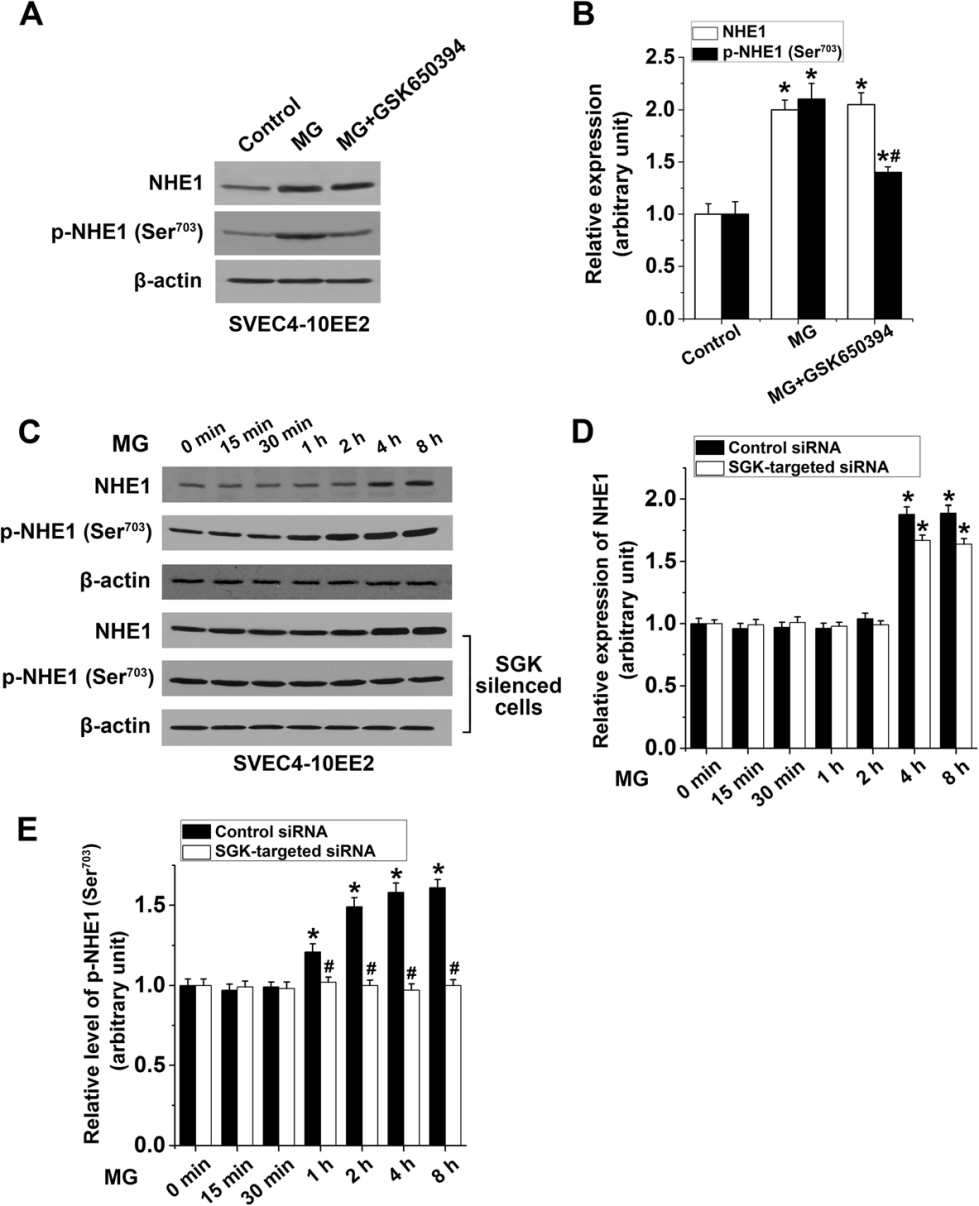
**Methylglyoxal-triggered endothelial SGK-dependent NHE1 activation. A** − **B**. Representative original Western blot **(A)** and means ± SEM (**B**; n = 4) showing total NHE1 (*white bars*) and phospho-NHE1 (14-3-3 binding motif at p-Ser^703^; *black bars*; relative to β-actin) determined in the absence (Control) or in the presence of MG-treatment (100 μM for 4 h) in EE2 ECs without (MG) or with the SGK1 inhibitor GSK650394 (20 μM, 1 h prior to MG). * indicates significant difference (p < 0.05) from the Control (0 μM MG). # indicates significant difference (p < 0.05) from MG alone. **C** − **E**. Representative original Western blot **(C)** and means ± SEM (n = 4) showing total NHE1 **(D)** and phospho-NHE1 (14-3-3 binding motif at p-Ser^703^; relative to β-actin; **E)** determined in MG-treated (100 μM for 0–8 h) EE2 ECs with scrambled siRNA (Control siRNA; *black bars*) or with SGK-targeted siRNA silencing (*white bars*). * indicates significant difference (p < 0.05) from 0 h. # indicates significant difference (p < 0.05) from scrambled control siRNA.

We then sought to elucidate the participation of SGK1 in the activation of endothelial NHE1 elicited by MG treatment. As shown in Figure 2A and B, MG treatment (100 μM) of EE2 ECs significantly upregulated total NHE1 and phospho-NHE1 as compared to the control. Treatment with the SGK1 inhibitor GSK650394 significantly attenuated MG-induced upregulation of phospho-NHE, but not total NHE1 (Figure 2A and B). To confirm the role of SGK1 in NHE1 activation, an additional series of experiments were performed using SGK-targeted siRNA silencing. As illustrated in Figure 2C and D, MG treatment significantly enhanced NHE1 protein expression in EE2 ECs at 4–8 h after MG treatment, an effect that was not significantly altered by SGK silencing. Furthermore, phospho-NHE1 levels in EE2 ECs were significantly enhanced at 1–8 h after MG treatment, an effect that was abolished by SGK silencing (Figure 2C and E). These data suggest that endothelial SGK1 activates NHE1 in response to MG treatment.

**Figure 3:**
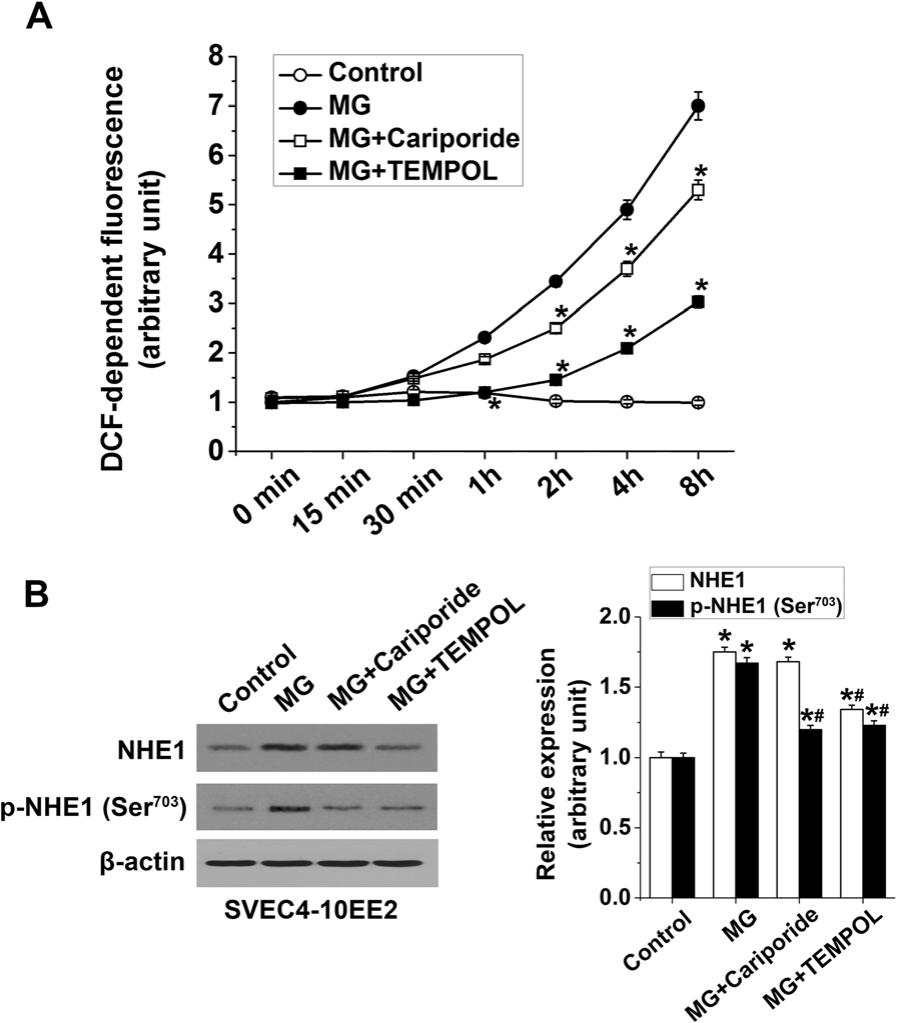
**Contribution of oxidative stress in methylglyoxal-triggered endothelial NHE1 activation. (A)** Means ± SEM (n = 6) of DCF-dependent fluorescence in EE2 ECs treated with MG (100 μM for 0–8 h) alone (MG) or co-treated with MG and cariporide (50 μM; 1 h prior to MG) or Tempol (300 μM; 1 h prior to MG). * indicates significant difference (p < 0.05) from MG alone. **(B)** Representative original Western blot and means ± SEM (n = 4) showing total NHE1 and phospho-NHE1 (14-3-3 binding motif at p-Ser^703^; relative to β-actin) determined in EE2 ECs treated with MG (100 μM for 1 h) alone or co-treated with MG and cariporide (50 μM; 1 h prior to MG) or Tempol (300 μM; 1 h prior to MG). * indicates significant difference (p < 0.05) from the absence of MG (Control). # indicates significant difference (p < 0.05) from MG alone.

To address the role of oxidative stress in MG-induced NHE1 activation, we analyzed ROS generation. Figure 3A shows that MG treatment significantly enhanced DCF-dependent fluorescence in a time-dependent manner, an effect that was significantly curtailed by treatment with cariporide or the antioxidant Tempol. These data indicate that NHE1 activation is required for MG-triggered ROS production. An additional series of experiments explored whether MG-induced ROS production [20] is essential as an upstream activator of endothelial NHE1. As shown in Figure 3B, total NHE1 expression in EE2 ECs was significantly upregulated after treatment with MG, an effect that was significantly blunted by the antioxidant Tempol but not by cariporide. In contrast, MG-triggered enhanced phospho-NHE1 expression was significantly reduced by both Tempol and cariporide treatment suggesting that MG-induced ROS production is crucial for NHE1 activation and both oxidative burst and NHE1 activation depend on each other in response to MG treatment (Figure 3B).

**Figure 4:**
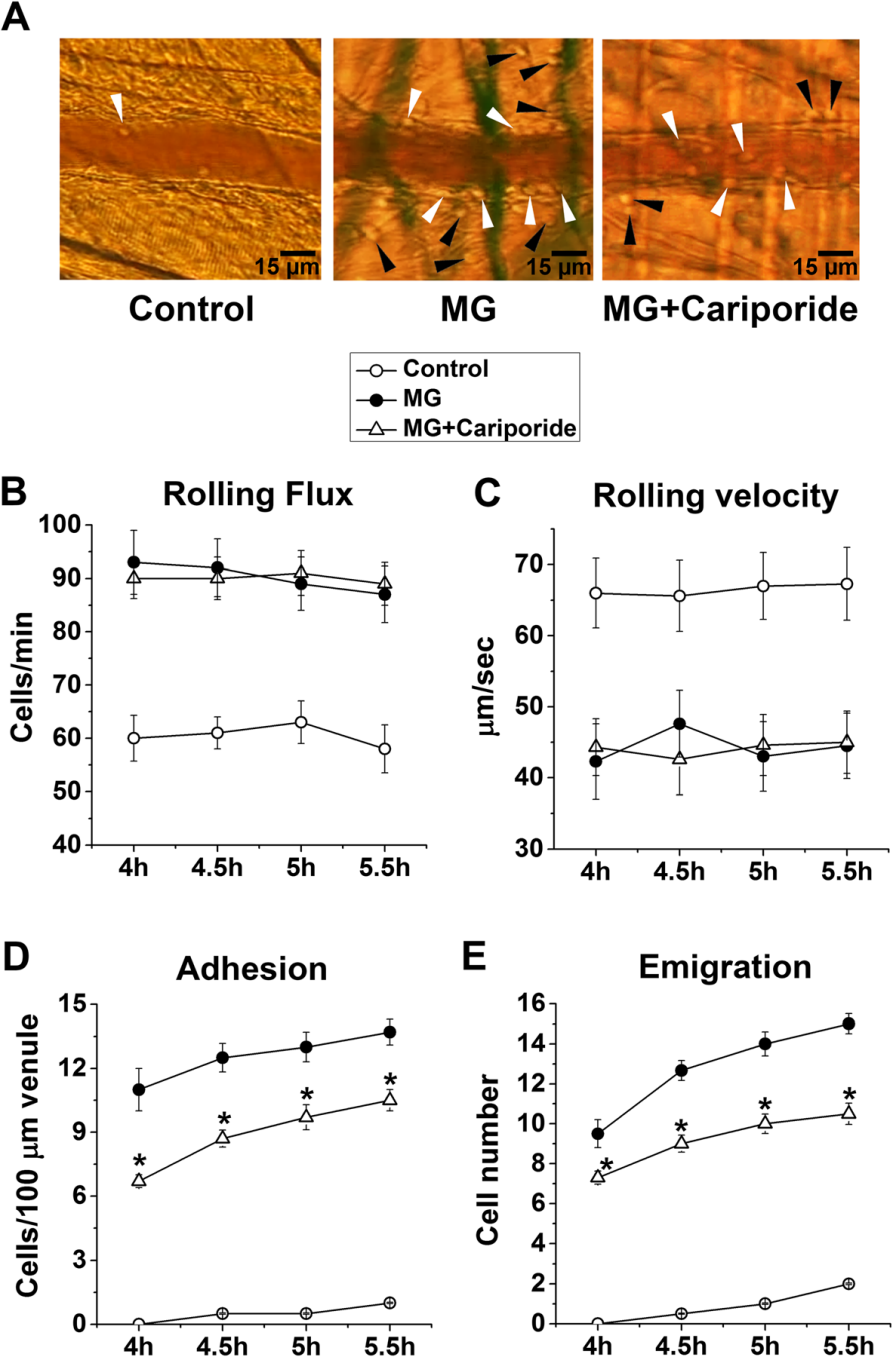
**Effect of cariporide on methylglyoxal-induced leukocyte recruitment** ***.*** **(A)** Representative intravital microscopy images showing adherent (*white arrowheads*) and emigrated (*black arrowheads*) leukocytes. **B** − **E**. Means ± SEM (n = 6) of leukocyte rolling flux (cells/min; **B)**, rolling velocity (μm/sec; **C)**, adhesion (cells/100-μm venule; **D)** and emigration (cells/443 × 286 μm^2^ field; **E)** determined 4.0 − 5.5 h after an intrascrotal injection of saline (Control) or MG (50 mg/kg) alone or co-treated with MG and cariporide (20 mg/kg, 1 h prior to MG). * indicates significant difference (p < 0.05) from MG alone.

Next, we explored whether SGK1-dependent NHE1 activation participates in MG-induced leukocyte recruitment. Intravital microscopy of murine cremasteric microvasculature revealed increased adhesion to endothelium and emigration of leukocytes following MG treatment, an effect that was thwarted by cariporide treatment (Figure 4). Analysis of leukocyte recruitment 4.0 − 5.5 h after an intrascrotal injection of MG showed decreased leukocyte rolling velocity and increased leukocyte rolling flux, adhesion and emigration as compared to saline control (Figure 4B-E). Pretreatment with cariporide, however, significantly blunted MG-induced increased leukocyte adhesion and emigration but did not modify leukocyte rolling flux and velocity (Figure 4B-E) indicating that NHE1 activation participates in leukocyte recruitment in response to excessive MG. Cariporide treatment reduced leukocyte adhesion by ~25% and emigration by ~34% at 5.5 h after MG treatment.

**Figure 5:**
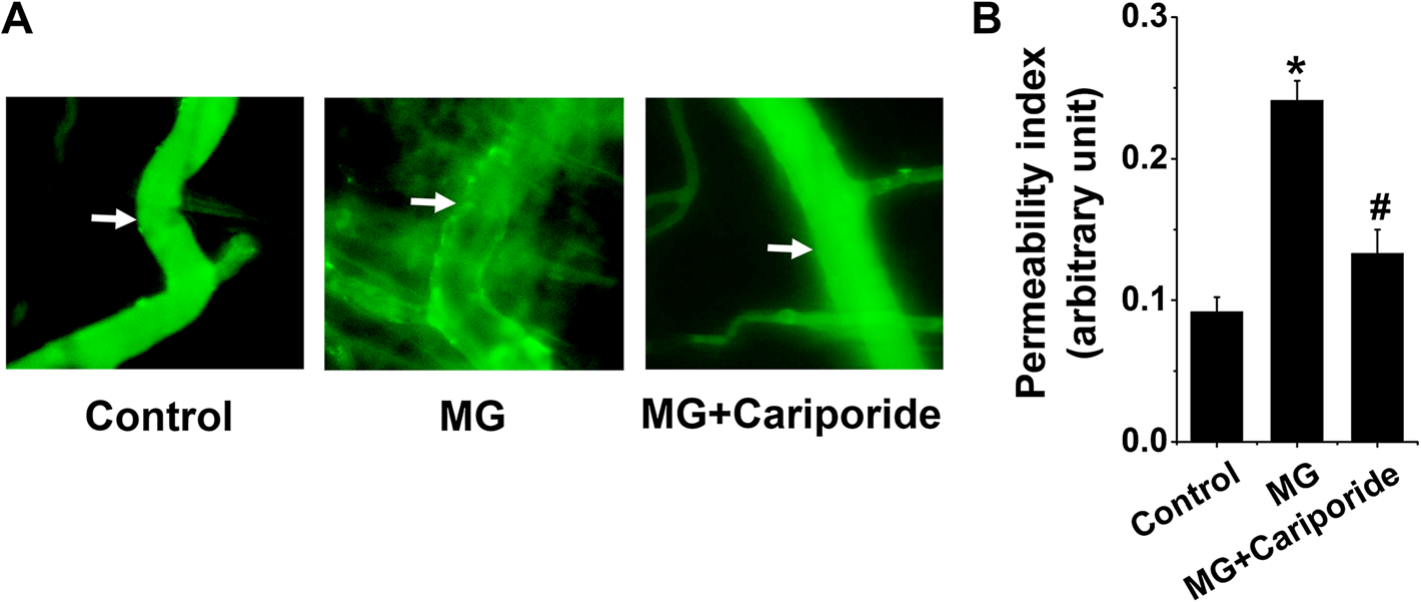
**Effect of cariporide on methylglyoxal-induced microvascular leakage. (A)** Representative fluorescence intravital microscopy images (arrows point to the segment of postcapillary venule where permeability index was determined) and **(B)** means ± SEM (n = 4) of permeability index analysis of mouse cremasteric postcapillary venules showing the leakage of FITC-conjugated BSA post 1-h i.v. injection and after an intrascrotal injection for 4 h with saline (Control), MG (50 mg/kg) alone or co-treatment with MG and cariporide (20 mg/kg, 1 h prior to MG). * indicates significant difference (p < 0.05) from the absence of MG (Control). # indicates significant difference (p < 0.05) from MG alone.

Impaired microvascular barrier function during leukocyte transendothelial migration fosters increased microvascular permeability. We, therefore, measured fluorescence changes of FITC-conjugated albumin inside and outside cremasteric postcapillary venules to examine the effect of cariporide treatment on MG-elicited microvascular leakage. As depicted in Figure 5, permeability index analysis revealed that an intrascrotal injection of MG increased microvascular permeability, an effect that was significantly inhibited by treatment with cariporide. These data indicate that NHE1 activation participates in MG-triggered increases in microvascular permeability.

**Figure 6:**
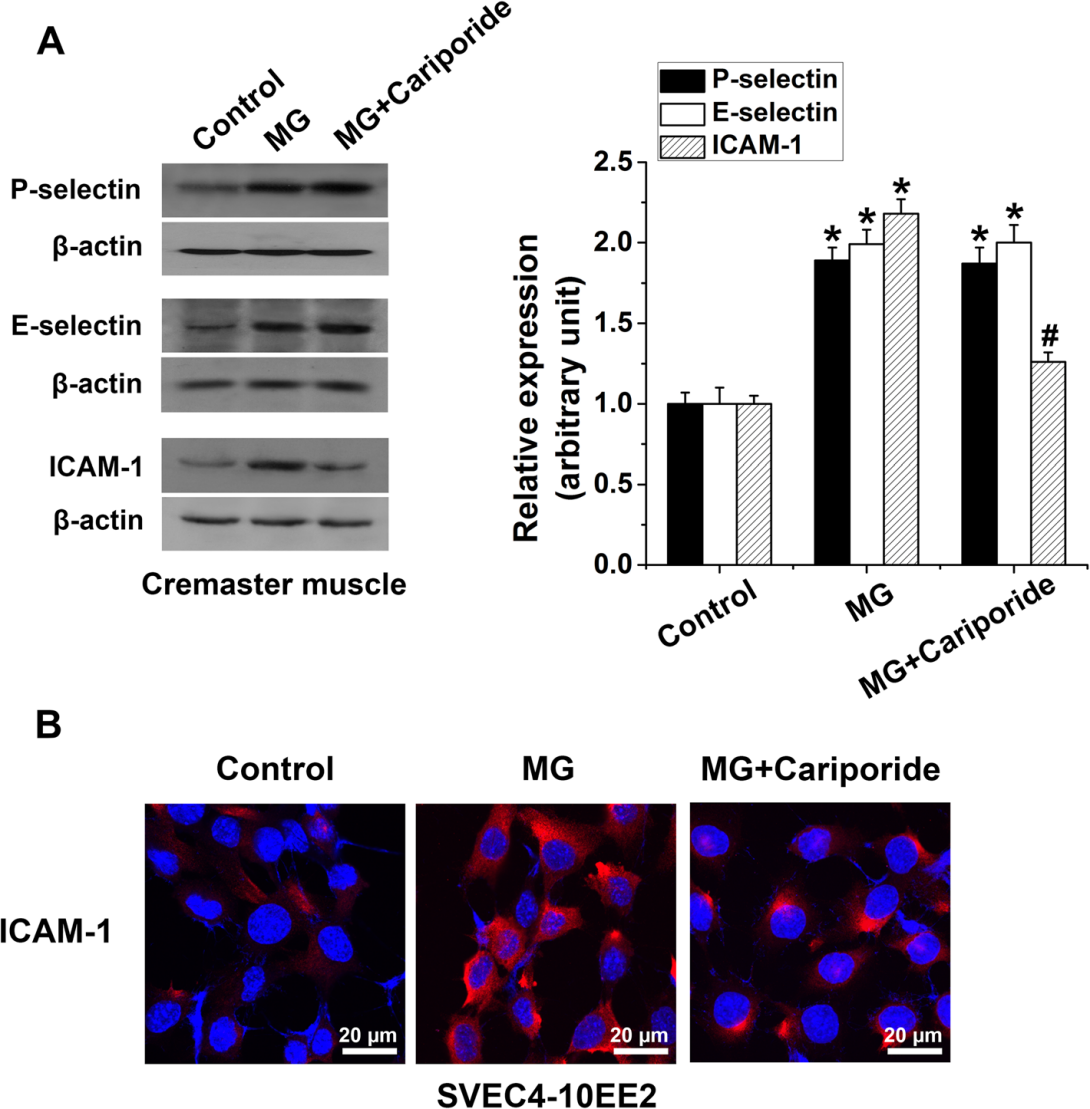
**Effect of cariporide on methylglyoxal-induced upregulation of endothelial adhesion molecule expression. (A)** Representative original Western blot and means ± SEM (n = 4) showing the expression of P-selectin, E-selectin and ICAM-1 (relative to β-actin) determined in whole cremaster muscle 4 h after an intrascrotal injection of saline (Control), MG (50 mg/kg) alone or co-treated with MG and cariporide (20 mg/kg, 1 h prior to MG). * indicates significant difference (p < 0.05) from absence of MG (Control). # indicates significant difference (p < 0.05) from MG alone. **(B)** Representative confocal micrographs of cell surface ICAM-1 immunostaining (*Red*) in EE2 ECs in the absence (Control) or in the presence of MG (100 μM, 4 h) alone or co-treatment with MG and cariporide (50 μM, 1 h prior to MG). Nuclear staining is shown in *blue* (Hoechst 33342).

To identify the mechanisms that regulate sensitivity of MG-induced leukocyte recruitment to cariporide, we analyzed the expression of endothelial adhesion molecules. Murine cremaster muscles treated with MG had enhanced expression of P- and E-selectins and ICAM-1. Cariporide treatment attenuated MG-induced ICAM-1 expression, but not P- and E-selectin expression *in vivo* (Figure 6A). To confirm these observations, we used confocal microscopy to visualize the surface expression of ICAM-1 on murine EE2 ECs. As shown in Figure 6B, MG treatment enhanced the expression of ICAM-1 on EE2 ECs, an effect that was blunted by cariporide treatment. These results confirm that inhibition of NHE1 in endothelial cells blunted MG-induced leukocyte adhesion and transmigration through the suppression of ICAM-1 upregulation indicating that MG-triggered activation of NHE1 regulates ICAM-1 upregulation and ICAM-1-mediated endothelial functions.

## Discussion

The present study reveals the role of SGK1-dependent activation of endothelial NHE1 in regulating leukocyte recruitment through the upregulation of ICAM-1 in response to MG. We observed that MG treatment potentiated dose- and time-dependent endothelial NHE1 mRNA and protein levels and triggered SGK1- and ROS-dependent NHE1 phosphorylation. By using intravital microscopy of cremasteric microvasculature in a previously described murine model of MG-triggered acute inflammation [19,21,22], we demonstrate that pharmacological inhibition of NHE1 using cariporide ameliorated leukocyte adhesion, transendothelial migration and microvascular leakage elicited by MG. Although, the decrease may not appear dramatic in terms of absolute recruited cell numbers presented in the data, it turns out to be a significant and stable difference in terms of inhibition of leukocyte recruitment *in vivo* which was determined in a small segment of the postcapillary venule. The physiological relevance of MG concentrations used in this study is corroborated by previous reports [49,50]. Intrascrotal injection of MG (50 mg/kg) was shown to yield MG concentrations of 1.7 μM and 3.9 nmol/mg protein in plasma and tissue respectively [19]. MG concentrations detected in cremaster muscle are similar to those detected in other organs [51,52]. Moreover, the concentration of MG (100 μM) used *in vitro* in the present study is based on previous reports [53-55].

Here, we show that MG treatment increased the levels of both total and phosphorylated endothelial NHE1. Mounting evidence suggests that NHE1 participates in the pathogenesis of various complications of diabetes. Recently, high glucose concentrations were demonstrated to stimulate p38 MAPK- and ERK1/2-dependent NHE1 activation in distal nephron cells [56]. Strikingly, hyperglycemia was shown to induce endothelial dysfunction via Ca^2+^-dependent calpain signaling mediated by the Na^+^/H^+^ exchanger [57]. In diabetic rats, vascular hypertrophy was accompanied by elevated NHE1 activity, an effect thwarted by cariporide administration [58]. Cariporide treatment was also found to ameliorate retinal microangiopathy in diabetic rats [59] and to curtail hyperglycemia-induced hypertrophy of rat cardiomyocytes [60]. Chronic cariporide administration was further shown to attenuate left ventricular hypertrophy in diabetic rats [61]. Intriguingly, cariporide treatment was shown to counteract the formation of methylglyoxal-derived advanced glycation end products (AGEs) in type 1 diabetic rats [62]. AGEs, in turn, are known to amplify inflammatory responses [63]. It is, therefore, tempting to speculate that enhanced MG levels may contribute to or even account for NHE1-mediated cardiovascular dysfunctions encountered in diabetic complications.

Mechanistically, PI3K signaling is decisive in NHE1-mediated cellular functions [64,65]. SGK1 is activated through PI3K and phosphoinositide-dependent kinase PDK1 [23,66]. The PI3K downstream kinase AKT was previously shown to phosphorylate and inhibit NHE1 [67]. Along these lines, SGK1 may play a decisive role in PI3K-dependent NHE1 regulation. It was reported that MG treatment did not alter SGK1 expression in neutrophils [22]. Accordingly, it is unlikely that cariporide treatment inhibits MG-induced leukocyte recruitment *in vivo* by mitigating neutrophil NHE1 activity. MG-induced leukocyte recruitment may, therefore, be effectively accomplished by activation of SGK1-dependent NHE1 activation in endothelial cells.

Redox imbalance is an essential pathological element of the diabetic milieu [68]. MG is known to stimulate ROS and superoxide production [16,20-22]. Furthermore, MG modulates the expression and functions of antioxidant cytoprotective molecules such as superoxide dismutase and H_2_S [69,70]. Compelling evidence links ROS-dependent signaling to NHE1 activation. Inhibition of NHE1 by cariporide was shown to blunt ROS formation in dendritic cells [40]. Conversely, antioxidant treatment is known to attenuate NHE activity [71]. Interestingly, ROS was previously shown to enhance NHE1 gene expression [72]. Thus, it is apparent from our data that ROS production and NHE1 activation upon stimulation with MG are dependent on each other.

Previous studies have shown a crucial role of NHE-dependent functions in the brain microvascular endothelium [36-38]. These studies, however, preclude us to draw mechanistic parallels of NHE-dependent functions in brain microvascular endothelial cells to peripheral endothelial cells due to their distinct features. Upregulation of adhesion molecules in diabetes is fostered by hyperglycemia [73-75] and increased levels of MG [19]. We observed that cariporide treatment did not alter selectin-dependent leukocyte rolling during MG-induced leukocyte recruitment *in vivo* but mitigated endothelial ICAM-1-dependent leukocyte adhesion. Surprisingly, thrombin-induced P-selectin upregulation and leukocyte rolling were reported to be inhibited by cariporide [76]. Cariporide treatment was shown to mitigate increased ICAM-1 mRNA levels in microvascular endothelial cells following ischemia-reperfusion [77] and to foster increased shedding of L-selectin on leukocytes [78]. Remarkably, cariporide treatment was found to decrease monocyte adhesion to endothelial cell, an effect that was attributed to decreased ICAM-1 expression [79]. Pharmacological blockade of NHE was previously reported to suppress NF-κB activation [80], an effect that contributes to reduced ICAM-1 expression [81]. Cariporide may, in addition, counteract inflammation by abating cytokine production [82].

MG was previously shown to trigger increased microvascular permeability [21], an effect mediated by oxidative stress [21,83]. Our data reveal that NHE1 inhibition attenuated MG-triggered microvascular hyperpermeability. However, whether NHE1 activation directly regulates microvascular barrier function is not known. It is possible that reduction in MG-triggered microvascular hyperpermeability after cariporide treatment results from the suppression of NHE1 activation which regulates both oxidative stress and transendothelial migration of leukocytes, key elements that are vital to the microvascular permeability regulation [83,84]. By the same token, leukocyte recruitment is also triggered by oxidative stress [21,85] and our data suggests that inhibition of MG-induced leukocyte recruitment by cariporide involves a redox-sensitive mechanism.

## Conclusions

**Figure 7:**
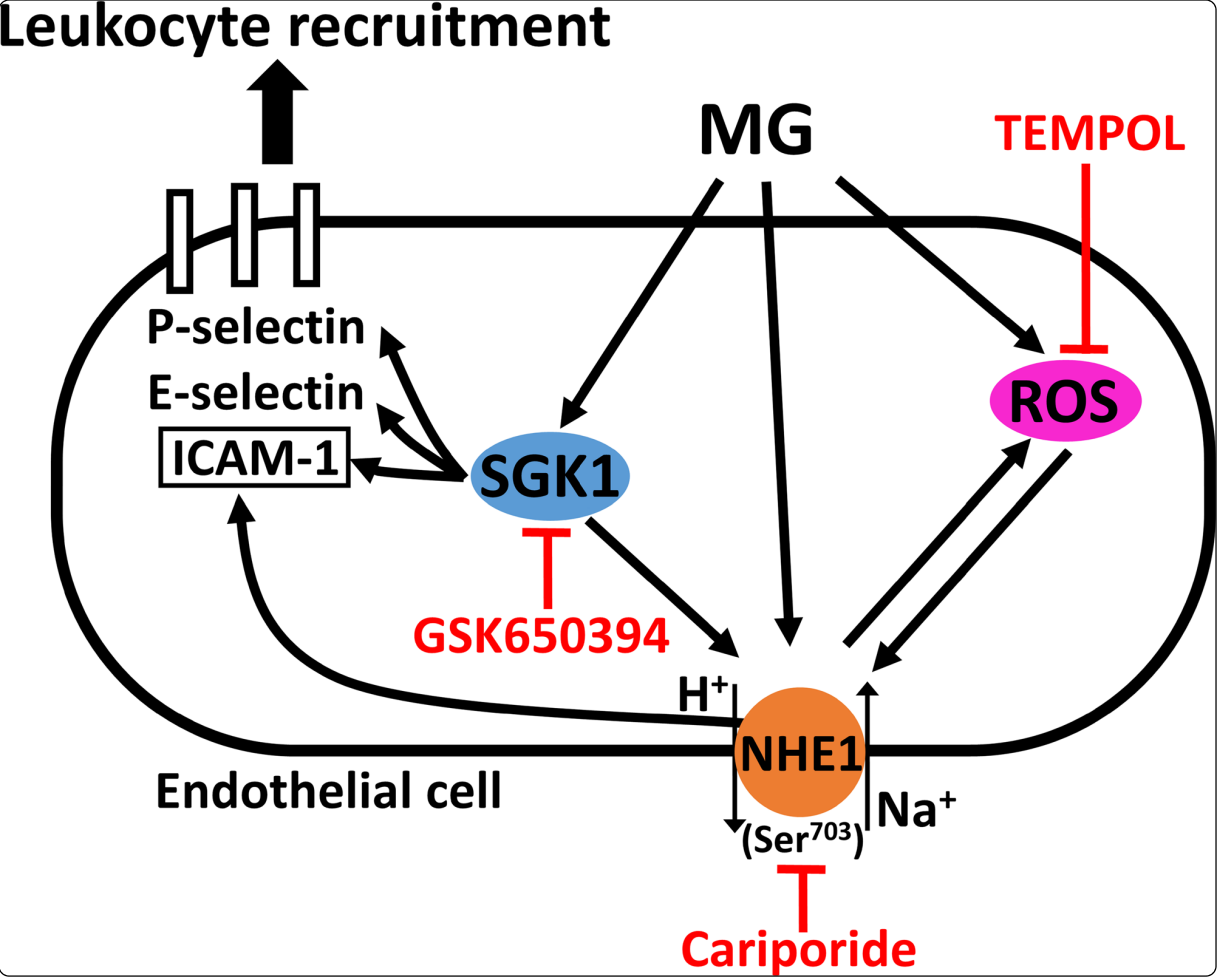
**Scheme of the role of Na** ^**+**^ **/H** ^**+**^ **exchanger NHE1 in methylglyoxal-induced leukocyte recruitment.**

In conclusion, MG elicits SGK1-dependent activation of endothelial Na^+^/H^+^ exchanger NHE1 which participates in MG-induced ROS production, upregulation of endothelial ICAM-1, leukocyte recruitment and microvascular hyperpermeability (Figure 7). Pharmacological inhibition of NHE1 attenuates the proinflammatory effects of excessive MG and may, thus, be beneficial in diabetes-associated inflammation.

## Abbreviations

MG: Methylglyoxal
Tempol: 1-oxyl-2,2,6,6-tetramethyl-4-hydroxypiperidine
ROS: Reactive oxygen species
PI3K: Phosphoinositide 3-kinases
SGK1: Serum- and glucocorticoid-inducible kinase 1
EE2 ECs: SVEC4-10EE2 endothelial cells
CREB: Cyclic AMP response element-binding protein
NF-κB: Nuclear factor-κB
NHE1: Na^+^/H^+^ exchanger 1
p38 MAPK: p38 mitogen-activated protein kinases
ERK1/2: Extracellular-signal-regulated kinases 1/2
ICAM-1: Intercellular adhesion molecule-1
AGEs: Advanced glycation end products
